# Determination of the complete genomic sequence of *Neofusicoccum luteum* mitovirus 1 (NLMV1), a novel mitovirus associated with a phytopathogenic *Botryosphaeriaceae*

**DOI:** 10.1007/s00705-017-3338-9

**Published:** 2017-04-27

**Authors:** Armelle Marais, Aurélia Nivault, Chantal Faure, Sébastien Theil, Gwenaëlle Comont, Thierry Candresse, Marie-France Corio-Costet

**Affiliations:** 1 UMR 1332 Biologie du Fruit et Pathologie, INRA, Univ. Bordeaux, CS 20032, 33882 Villenave d’Ornon Cedex, France; 2 UMR 1035 Santé et Agroécologie du Vignoble, INRA, Bordeaux Sciences Agro, CS 20032, 33882 Villenave d’Ornon Cedex, France

## Abstract

*Neofusicoccum luteum* species belongs to the *Botryosphaeriaceae* family and is involved in grapevine wood decay diseases. The present study reports the discovery and the molecular characterization of a novel mitovirus infecting this fungus. Double-stranded RNAs were purified from cultivated *N. luteum* and analysed by next generation sequencing. Using contigs showing BlastX homology with the RNA-dependent RNA polymerase (RdRp) gene of various members of the family *Narnaviridae*, a single contig of approximately 1.2 kb was constructed. The genomic sequence was completed and phylogenetic analyses indicated that this virus represents a new member of the genus *Mitovirus*, for which the name of “*Neofusicoccum luteum mitovirus 1*” is proposed. The genome is 2,389 nucleotides long and, based on the fungal mitochondrial genetic code, it encodes a putative protein of 710 amino acids, homologous to the RdRps of members of the *Narnaviridae* family. The neofusicoccum luteus mitovirus 1 (NLMV1) RdRp contains the six conserved motifs previously reported for mitoviral RdRps. Our findings represent the first evidence that a mycovirus can infect *N. luteum*, an important pathogenic fungus of grapevine.

**Electronic supplementary material:**

The online version of this article (doi:10.1007/s00705-017-3338-9) contains supplementary material, which is available to authorized users.

## Introduction

Since the first discovery of viruses in *Agaricus bisporus* [1], mycoviruses have been described in all major taxa of fungi. Most of them have either double-stranded or single-stranded RNA genomes, and a few have a DNA genome [2]. With the expansion of novel sequencing technologies, the number of fungal viruses identified has dramatically increased in the last couple of years. Mitoviruses seem to be widespread among phytopathogenic fungi and many putative new viral species of the genus *Mitovirus* have been proposed [3–6]. Taxonomically, mitoviruses are members of the family *Narnaviridae*, which comprises the genera *Mitovirus* and *Narnavirus*. They are considered as the simplest mycoviruses, in that they contain a single linear molecule of positive-sense RNA, with a single open reading frame (ORF) encoding the viral RNA-dependent RNA polymerase (RdRp) in the mitochondrial genetic code. Mitoviruses do not form particles, but form lipid membrane-bound vesicles, and are confined to the mitochondria of their host cells, in contrast to narnaviruses, which replicate in the cytosol [7]. Several mycoviruses, including some mitoviruses [7, 8] have been described to confer hypovirulence to their phytopathogenic fungal host, providing a way to develop biocontrol strategies [2].

*Neofusicoccum luteum* is involved in Botryosphaeria dieback, a worldwide grapevine trunk disease [9]. In the present study, we report the discovery of a novel species of mitovirus infecting *N. luteum* for which the name of “*Neofusicoccum luteum mitovirus 1*” is proposed. The complete genomic sequence of neofusicoccum luteum mitovirus 1 (NLMV1) was determined and consists of a 2,389 nucleotides long RNA molecule encoding a putative RdRp of 710 amino acids (aa).

## Virus material

The *N. luteum* isolate used was NL-37 (GWEMA-UMR SAVE collection), sampled in a vineyard in 1996, on canes of *Vitis vinifera*. The fungus was grown as described previously [10].

## Results

Double-stranded RNAs were purified and analyzed on agarose prior to random amplification and Miseq sequencing in a multiplexed scheme as previously reported [11]. After quality trimming and demultiplexing, a total of 136,741 paired reads were obtained, assembled using home-made pipelines, and compared to GenBank database using BlastN and BlastX. A contig of approximately 1.2 kb, integrating 2.3% of the total reads, revealed significant similarities with various members of the genus *Mitovirus*. The 5’ and 3’ genome ends were determined using purified dsRNA as template and 5’ and 3’ Rapid Amplification of cDNA Ends (RACE) with internal primers designed from the contig, following the kit manufacturer’s instructions (Takara Bio Europe/Clontech, Saint-Germain-en-Laye, France).

**Fig. 1:**
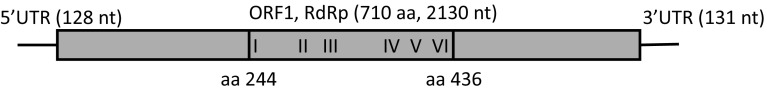
Schematic representation of the genome organization of neofusicoccum luteum mitovirus 1. The open reading frame 1 (ORF1) encoding the RNA-dependent RNA polymerase is shown as the rectangular box, as well as the conserved region with the six conserved motifs typically observed in mitoviral RdRps (I, II, III, IV, V, and VI). The amino acid position of the start and end of the conserved region is also shown. UTR, Untranslated region

**Fig. 2:**
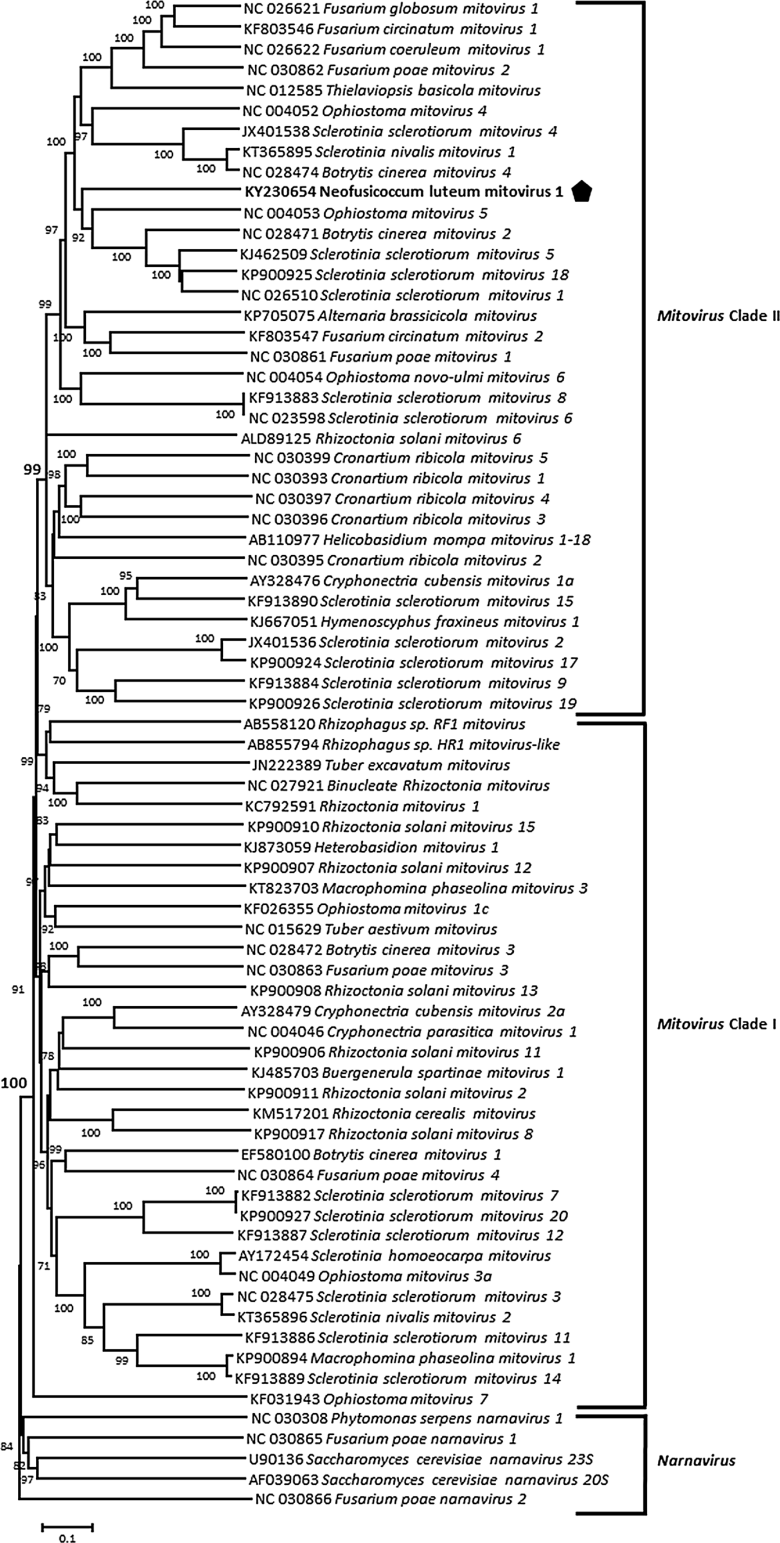
Neighbor-joining phylogenetic tree reconstructed using the complete amino acid sequences of RNA-dependent RNA polymerases from members of the family *Narnaviridae*. The tree uses strict identity distance and the statistical significance of branches was evaluated by bootstrap analysis (1,000 replicates). Only values higher than 70% are indicated. The scale bar represents 10% amino acid divergence. The sequence of the neofusicoccum luteum mitovirus 1 is marked by a black pentagon

The complete NLMV1 genome sequence is 2,389 nt long (GenBank accession number KY230654), which is a typical size for a mitoviral genome [12]. Its GC content is relatively low (30.6%), which is also characteristic of members of the genus *Mitovirus*. The genetic organization of the genome is shown in Fig. 1. It contains a 128 nt long 5’-untranslated region (UTR) and a 3’-UTR of 131 nt, and, as most mitoviruses, has no 3’ poly(A) tail. As expected for a mitovirus, the 5’- and 3’-UTRs of NLMV1 could be folded into two stable stem-loop structures with ∆*G* values of -35.7 kcal/mol and -52.3 kcal/mol, respectively. In contrast, no inverted complementarity between the two UTRs was detected, suggesting absence of a panhandle structure. This has been described in other mitoviruses, such as alternaria arborescens mitovirus 1 [6]. Using the mitochondrial genetic code, a single large ORF was detected, encoding a 710 aa long polypeptide, sharing significant but weak sequence similarity with the RdRp gene of several mitoviruses. The most closely related species was *Sclerotinia sclerotiorum mitovirus 18*, with 38.4% of aa sequence identity. Considering the species demarcation criteria accepted for the genus *Mitovirus* (less than 40% of aa identity in the RdRp gene, [12]), NLMV1 should be considered as a member of a new species within this genus. A neighbor-joining tree was reconstructed with the RdRp sequences of members of the family *Narnaviridae* (Fig. 2) and showed that NLMV1 clusters with the Clade II of mitoviruses. As expected, analysis of the conserved domains in the NLMV1 RdRp revealed the presence of the six motifs (I to VI) which are typical of mitoviral RdRps [13].

Some mitoviruses have been shown to affect the virulence of their hosts [8]. This study is the first report of a mitovirus infecting *N. luteum* involved in grapevine Botryosphaeria dieback. Further studies are needed to assess the virulence of NLMV1, and to explore the overall diversity of viruses infecting this pathogenic fungus.

## Electronic supplementary material

Below is the link to the electronic supplementary material.

Supplementary material 1 (TXT 2 kb)
